# Enniatin B1: Emerging Mycotoxin and Emerging Issues

**DOI:** 10.3390/toxins15060383

**Published:** 2023-06-06

**Authors:** Beatrice De Felice, Leon J. Spicer, Francesca Caloni

**Affiliations:** 1 Department of Environmental Science and Policy (ESP), Università degli Studi di Milano, Via Celoria 10, 20133 Milan, Italy; beatrice.defelice@unimi.it; 2 Department of Animal and Food Sciences, Oklahoma State University, Stillwater, OK 74078, USA; leon.spicer@okstate.edu

**Keywords:** emerging mycotoxins, enniatin B1, biological characteristics, toxicological effects

## Abstract

Although over the last 10 years several studies have focused on the emerging mycotoxins known as enniatins (ENNs), there is still a lack of knowledge regarding their toxicological effects and the development of a correct risk assessment. This is especially true for enniatin B1 (ENN B1), considered the younger sister of the widely studied enniatin B (ENN B). ENN B1 has been found in several food commodities and, as with other mycotoxins, presents antibacterial and antifungal properties. On the other hand, ENN B1 has shown cytotoxic activity, impairment of the cell cycle, the induction of oxidative stress, and changes in mitochondrial membrane permeabilization, as well as negative genotoxic and estrogenic effects. Overall, considering the paucity of information available regarding ENN B1, further studies are necessary to perform a risk assessment. This review summarizes information on the biological characteristics and toxicological effects of ENN B1 as well as the future challenges that this mycotoxin could present.

## 1. Introduction 

**Figure 1:**
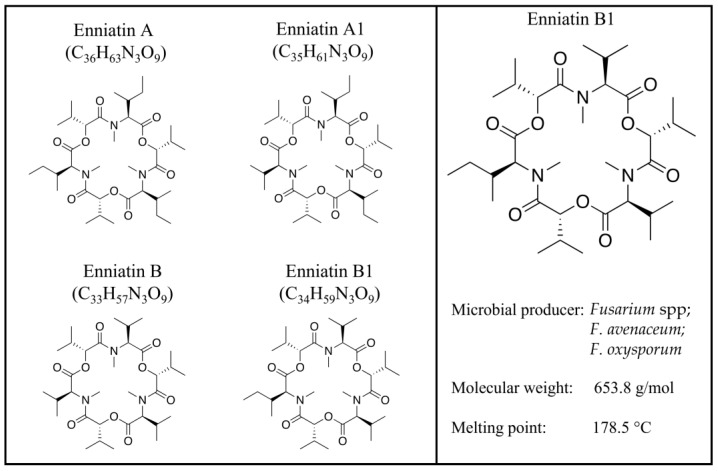
Enniatin A, A1, B, and B1 structures.

*Fusarium* mycotoxins, secondary metabolites of fungi, are now identified as traditional mycotoxins, such as fumonisins, trichothecenes, zearalenone, and emerging ones, which are neither routinely determined nor legislatively regulated [1,2] and whose interest is always growing for their occurrence and impact, as well as the need to finally complete a correct risk assessment [3]. Fumonisins, thricothecens, and zearalenone are considered the most widespread and toxic fusariotoxins, negatively affecting humans and animals [3]. In general, their toxic effects, acute or chronic, are carcinogenic, mutagenic, teratogenic, immunosuppressive, hepatotoxic, neurotoxic, and reproductive [2,3]. Beauvericin (BEA) and enniatins (ENNs); cyclic hexadepsipeptides consisting of alternating D-α-hydroxy-isovaleryl-(2-hydroxy-3-methylbutanoic acid) and amino acid units; fusarin C (FUC), which belongs to the fusarin family; fusaproliferin (FUS), a bicyclic sesterterpene consisting of five isoprenic units; and moniliformin (MON), a 3-hydroxycyclobut-3-ene-1,2-dione, are emerging fusariotoxins mainly produced by *Fusarium avenacum*, *Fusarium verticillioides*, *Fusarium proliferaturm*, and *Fusarium subglutinans* [4]. Although the first enniatin (ENN) was isolated many years ago [5], these mycotoxins have gained more and more attention over the last 10 years, as they are considered natural contaminants of emerging concern, and a correct evaluation of their toxicological risks remains to be performed [6,7]. ENNs—which include different analogs, including ENN A, ENN A1, ENN B, and ENN B1 [8] (Figure 1)—are mainly produced by *Fusarium* spp. [8,9], whose level of contamination is known to be influenced by multiple factors [10,11,12]. ENNs have many of the classical characteristics of traditional mycotoxins, are naturally present in several feed and food commodities worldwide [13,14,15,16,17], and are often detected in combination with each other or with other mycotoxins [2,17,18,19]. ENNs, lipophilic and ionophoric molecules [8], exert a two-faced Janus action:on one side, they exhibit antibacterial, anthelmintic, antiviral, insecticidal, fungicidal, herbicidal, and anticancer properties [8,20]. In vitro studies demonstrate the toxicological effects of ENNs, ranging from cytotoxicity [8] to reproductive effects [21], showing similar behavior to the multifaced BEA [22].

## 2. Enniatin B1

Although ENN B1 has been less studied than ENN B [8], ENN B1 has emerged as one of the more relevant [9] and prevalent contaminants in commodities among ENNs [17,23,24]. Specifically, the occurrence of ENN B1 is usually related to cereal crops, such as barley, maize, oats, rye, rice, triticale, and wheat [25]. Even if cereal crops are the most often contaminated, ENN B1 can also occur in other commodities such as potatoes, eggs, apples, peanuts, soybeans, and rapeseed [25]. The occurrence of ENN B1 appears to be worldwide and, indeed, was detected in European (i.e., Italy, Spain, Denmark, France, Norway, Finland, Sweden, Belgium, Germany, Poland, Romania, and Serbia), North American (i.e., Canada), Asian (i.e., China and Iran), and African (i.e., Morocco, Cameroon, Tunisia, Egypt, and Mozambique) countries [25]. This global-scale occurrence of ENN B1 suggests that this mycotoxin does not have a specific climatic condition in which it occurs and, therefore, represents a global health problem. However, with expected changes in climate such as rising temperatures and modifications in the pattern of rainfall events, it is not possible to exclude the idea that these climate changes will modify ENN B1 occurrence and its possible toxicity. Even if the climate presents a strong influence on ENN occurrence, it is important to consider how the site-specific and crop-specific differences could be related to other factors such as biology, the environment, harvest, and storage, as well as distribution factors. The biological factors affecting the occurrence of ENNs and specifically ENN B1 include how susceptible a specific crop is or how compatible a specific crop is with the contamination of the fungus. Environmental factors include temperature, moisture, and the presence of predators (i.e., insects or birds). In addition, the way in which the crop is harvested, as well as how it is stored, can influence the occurrence of mycotoxins [9,26]. Considering their widespread presence in commodities, mycotoxins and, especially, ENN B1 are easily ingested by both animals and humans. Nevertheless, to date, there is no legislation regarding these contaminants. The most recent document analyzing this issue is a scientific opinion released by the European Food Safety Authority (EFSA) in 2014, highlighting that acute exposure to ENNs is not a concern for human health, and further studies should be a priority to investigate possible chronic toxicity. A similar outcome appears for the toxicological data regarding livestock and companion animals since no data are available regarding the possible toxicity of ENNs in these organisms. Even if this document is a first step toward the development of scientific legislation for ENNs, there is still a great limitation because of the paucity of information available regarding these emerging contaminants. 

Therefore, the literature is lacking, and through this review, the authors want to take stock of the knowledge related to this emerging mycotoxin, considering both biological actions and toxicological effects, in order to direct research for a correct risk assessment.

## 3. Biological Characteristics 

**Figure 2:**
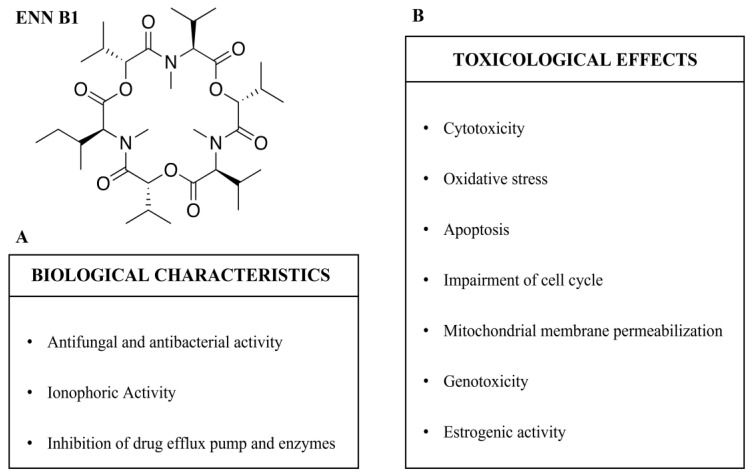
Enniatin B1 (ENN B1): biological characteristics (**A**) and toxicological effects (**B**).

ENN B1 presents several different biological characteristics, most of which are in common with other ENNs derived from *Fusarium* spp. [8,9]. The biological characteristics of ENN B1 are summarized in Figure 2A and Table 1.

### 3.1. Antifungal and Antibacterial Activity 

Moderate antifungal activity in ENN B1 against *Candida albicans*, *Cryptococcus neoformans*, and *Mycobacterium intracellulare* was highlighted by Chiminelli et al. [21]. Moreover, antifungal activity was demonstrated against the plant pathogen *Eutypa armeniacae* [27]. However, in a recent study performed by Meca et al. [28], there was a lack of ENN B1 antifungal activity (ranging from 0.1 to 20 μg) against *Fusarium verticilloides, Fusarium sporotrichioides*, *Fusarium tricinctum*, *Fusarium poae, Fusarium oxysporum*, *Fusarium proliferatum*, *Beauveria bassiana*, *Trichoderma harzianum*, *Aspergillus flavus*, *Aspergillus parasiticus*, *Aspergillus fumigatus*, *Aspergillus ochraceus*, and *Penicillium expansum.* Besides antifungal activities, ENN B1 acts as an antibacterial agent against some human pathogenic bacteria such as *Escherichia coli*, *Yersinia enterocolitica*, *Clostridium perfringens*, and *Enterococcus faecium* [29]. The antibacterial activity of ENN B1 was confirmed against *Bifidobacterium adolescentis* at doses ranging from 20 ng to 20,000 ng, as well as against *Streptococcus thermophilus*, 2 strains of *Lactobacillus*, and 2 other strains of *Bifidobacterium* [30], while antibacterial activity was absent against *Bacillus subtilis* strains and 20 out of 22 *Saccharomyces cerevisiae* strains [30]. In addition, ENN B1 mixed with other ENNs (i.e., ENN B and ENN B4) showed antibacterial activity against *Mycobacterium tuberculosis* [31]. On the other hand, several studies have shown that ENNs are also phytotoxic compounds [29,30]. Specifically, a study by Herrmann et al. [32] highlighted that a mixture of ENN A, A1, B, and B1 (ratio 5:15:35:45) caused necrotic damage to potato tuber tissue at 50 and 100 µg/slice. Lastly, although insecticidal and anthelmintic activity has been confirmed for different ENNs [8,33], to date, no information is present regarding the possibility of insecticidal or anthelmintic activity in ENN B1. However, ENN B1 was found to be cytotoxic in a mixture with other ENNs (A, A1, and B) against the *Spodoptera frugiperda* cell line, an insect cell line used to investigate the in vivo cytotoxicity of fungal metabolites [34].

### 3.2. Ionophoric Activity

In addition to antifungal and antibacterial activity, one of the best-known biological characteristics of ENNs is their ionophoric activity [8,35]. Indeed, ENNs incorporate easily into cell membranes as a passive channel by forming cation-selective pores (K^+^, Na+, and Ca^2+^), which can affect cell homeostasis by changing the intracellular ion concentration [35]. However, data regarding the ionophoric activity of ENN B1 are scarce since, to date, most studies have focused on ENN A and B or BEA [20,35,36]. Previously, when ENN B1 was compared with ENN B and A1, lower activity was registered [37,38]. Additionally, ENN B1 was recently reported to permeabilize the lysosomal membrane by destabilizing the LAMP-2 complex at a concentration close to the EC_50_ (1.5–1.7 µmol/L) [39]. Moreover, a recent study highlighted the ability of ENN B1 to alter calcium homeostasis leading to apoptotic cell death in SH-SY5Y human neuroblastoma cells [40].

### 3.3. Inhibition of Drug Efflux Pump and Enzymes

Some biological characteristics of ENNs such as the inhibition of drug efflux pumps and the inhibition of enzymes have stimulated interest in these molecules for use in medicine. ENNs were proven to inhibit ABC (ATP-binding cassette) transporters and, therefore, have started to be considered for their utility in cancer therapy [41]. Indeed, inhibiting ABC transporters causes a decrease in drugs (i.e., chemotherapeutics) transported out of cells [41]. In addition, ENN B1 was found to be a potent and specific inhibitor of a functional homolog of mammalian P-glycoprotein (i.e., pleiotropic drug resistance 5 protein—Pdr5p), the latter of which is one of the causes of multidrug resistance in tumors [42,43]. Additionally, in a different cancer-related study, ENN B1 was found to have strong apoptotic activity and disrupted extracellular-regulated protein kinase (ERK), a protein associated with cell proliferation [35]. Furthermore, the induction of apoptosis was reported for H4IIE cells incubated in ENN B1 (1 µm) for 24 h [8]. Lastly, ENN B1 was reported to inhibit the enzyme acyl-CoA:cholesterol acyltransferase [35] and, therefore, may be important in atherosclerosis and hypercholesterolemia therapies.

**Table 1:** ENN B1 biological characteristics.

Characteristics	Enniatins	Toward/Activity	Ref.
Antifungal activity	ENN B1	*Candida albicans;* *Cryptococcus neoformans;* *Mycobacterium intracellulare.*	[21]
	ENN B1	*Eutypa armeniacae*	[27]
	ENN B1	*Escherichia coli;* *Yersinia enterocolitica;* *Clostridium perfringens;* *Enterococcus faecium.*	[29]
Antibacterial activity	ENN B1	*Bifidobacterium adolescentis;* *Streptococcus thermophilus;* *Lactobacillus* (2 strains); *Bifidobacterium* (2 strains).	[30]
	ENN B1, B, B4	*Mycobacterium tuberculosis*	[31]
	ENN B1	Affects cell homeostasis by changing the intracellular ion concentration.	[35]
Ionophoric activity	ENN B1	Permeabilization of the lysosomal membrane.	[39]
	ENN B1	Alteration of calcium homeostasis.	[40]
	ENN B1	Inhibition of ABC (ATP-binding cassette) transporters.	[41]
Inhibition of drug efflux pumps and enzymes	ENN B1	Inhibition of pleiotropic drug resistance 5 protein—Pdr5p.	[42,43]
	ENN B1	Disruption of extracellular-regulated protein kinase (ERK).	[35]
	ENN B1	Inhibition of enzyme (i.e., acyl-CoA:cholesterol acyltransferase).	[35]

## 4. Toxicological Effects

The toxicity of ENN B1 was investigated with different cell lines in in vitro studies both individually [28,36,44] and in mixtures with other ENNs [45,46]. Similar to other ENNs (i.e., ENN A, ENN A1, and ENN B), ENN B1 was found to present cytotoxic activity [28,44,45] that impaired the cell cycle [47,48] and induced an oxidative stress situation that can lead to apoptosis [49,50,51], and it induced changes in mitochondrial membrane permeabilization [36,52]. Moreover, recently, the genotoxic and estrogenic activity of ENN B1 was suggested [53,54]. Conversely, compared with the availability of several in vitro studies, to date, the number of in vivo studies focused on ENN B1 is still scant. The toxicological effects of ENN B1 are summarized in Figure 2B and Table 2.

### 4.1. Cytotoxicity

As stated earlier, different studies have highlighted how the cytotoxicity of ENNs could be related to their ionophoric characteristics and, therefore, connected to lysosomal destabilization, as well as mitochondrial permeabilization [8]. The dose-dependent cytotoxicity of ENN B1 was confirmed in vitro using human epithelial colorectal adenocarcinoma (CaCo-2, [51]), human colon carcinoma (HT-29 [28]), intestinal porcine epithelial (IPEC-J2 [55]), human liver carcinoma (HepG2 [56]), human fibroblast-like (MRC-5 [56]), and Chinese hamster ovary (CHO-K1 [48]) cells. Different IC_50_ values were found for the different cell lines, with the CHO-K1 cells being the most sensitive to exposure to ENN B1. Specifically, exposure times ranged from 24 h to 72 h, and the reported IC_50_ ranged between 10.8 µM to 0.8 µM for Caco-2 cells [51], between 16.6 µM and 3.7 µM for HT-29 cells [28], between 24.3 µM and 8.5 µM for HepG2 cells [56], between 4.7 µM and 4.5 µM for MRC-5 [56], and between 4.53 µM to 2.47 μM for CHO-K1 [46]. On the other hand, to date, no clear information is available regarding IPEC-J2 EC_50_ for ENN B1. However, regarding IPEC-J2 cells, a reduction in transepithelial electrical resistance, an indicator of barrier integrity, was noted after exposure to 5 µM of ENN B1, but no reduction in cell viability was noted [55]. Lastly, the cytotoxic activity of ENN B1 was also confirmed for other cell lines such as insect SF-9 cells with an IC_50_ of 6.6 µM after 48 h of exposure [34] and porcine kidney (PK-15) cells with an IC_50_ of 41 µM after 24 h of exposure. Collectively, the cytotoxic activity of ENN B1 ranges from 0.8 µM to 41 µM. Compared with other ENNs, ENN B1 presents higher cytotoxicity than ENN B but lower toxicity to that of ENN A and A1 in proliferating IPEC-J2 after 24 h of incubation [57]. On the other hand, Novak et al. [58] reported that ENN B1 cytotoxicity was lower than that of ENN A and ENN B but higher than that of ENN A1 after 48 h of incubation. Lastly, several studies investigated the cytotoxicity of various mixtures of ENNs [45,46]. Lu et al. [46] investigated the cytotoxicity of binary and tertiary combinations of different ENNs using CHO-K1 cells and discovered an IC_50_ of 0.44 ± 0.15 µM for a mixture of ENN A1 + B and 0.97 ± 0.48 µM for a mixture of ENN A1 + B + B1. Furthermore, an additive effect was noted for the binary mixture (i.e., ENN A + ENN B1 and ENN B + ENN B1), while a synergistic effect was noted for the tertiary mixture (i.e., ENN A + ENN A1 + ENN B1; ENN A + ENN B + ENN B1; and ENN A1 + ENN B + ENN B1) [46]. Interestingly, synergy was recorded at higher a concentration of ENN A for both the binary and tertiary mixtures, while an antagonistic effect was recorded at lower concentrations of ENN A for the tertiary mixture [46]. The cytotoxicity of the mixtures was investigated using Caco-2 cells treated with concentrations ranging from 0.9 to 15.0 µM [45]. Specifically, a synergistic effect was observed for Caco-2 cells exposed to a binary mixture of ENN A1 + ENN B1, while an additive effect was observed for a tertiary mixture of ENN A + ENN A1 + ENN B1, ENN A1 + ENN B + ENN B1, and ENN A + ENN B + ENN B1, as well as for a quaternary mixture of ENN A + ENN A1 + ENN B1 + ENN B [45]. In contrast, an antagonistic effect was recorded for a binary mixture of ENN B1 + ENN B [45]. Lastly, Kolf-Clauw et al. [59] investigated the possible cytotoxicity of ENN B1 in mixtures with other fusariotoxins such as trichothecenes and found that the combination of these toxins led to an antagonistic effect and the down-modulation of gastrointestinal toxicity in IPEC1 cells.

### 4.2. Oxidative Stress

The overproduction of reactive oxygen species (ROS) is one of the key factors involved in the onset of oxidative stress caused by ENNs [8,49,51]. Regarding ENN B1, the ability to induce oxidative stress was confirmed in CaCo-2 cells exposed to concentrations of 1.5 and 3 µM by Prosperini et al. [51]. Moreover, the overproduction of ROS in CaCo-2 cells involved oxidative damage, including lipid peroxidation, DNA damage, and necrosis [51]. Additionally, intracellular ROS generation in mouse blastocysts was reported at concentrations of 1–10 µM of ENNs during embryo development [60]. Lastly, a recent study performed by Cimbalo et al. [49] investigated the acute effects of an 8 hr exposure to a mixture of ENNs (ENN A, ENN A1, ENN B and ENN B1) in Wistar rats, highlighting that these mycotoxins can induce mitochondrial disorders and induce oxidative stress in intestinal barrier functions. Conversely, no ROS overproduction was found in SH-SY5Y human neuroblastoma cells treated with 0.1 µM and 10 µM of ENN B1, but ROS production increased with ENNA1 [61].

### 4.3. Apoptosis

Several studies report that apoptosis mediated by ENNs is primarily connected to ROS overproduction [8,50,60]. In addition, the apoptotic effect caused by exposure to ENNs may involve caspase enzymes [61]. Huang et al. [50] showed that exposure to ENN B1 triggered ROS overproduction, leading to the activation of caspase-3 and caspase-9 and subsequently apoptosis in mouse blastocysts. Moreover, Wang et al. [47] suggested that ENN B1 (10, 25, and 50 μM) was able to induce apoptosis in pig embryos by destroying the anti-apoptosis signaling pathway. Indeed, ENN B1 exerted its toxic effects by upregulating the transcription of the proapoptotic genes *Bax* and *Caspase3* and downregulating the expression of the antioxidant genes *Sod1*, *Gpx4*, and *Cat* and the antiapoptotic factor *Bcl2l1* [47]. In SH-SY5Y human neuroblastoma cells, the alteration of Ca^2+^ homeostasis induced by ENN B1 (0.1 µM and 10 µM) led to caspase-induced apoptotic cell death [47]. In contrast, no changes in apoptosis were found in HepG2 cells treated with ENN B1 (1.5 and 3 μM) for 24, 48, and 72 h [48].

### 4.4. Impairment of Cell Cycle

Several studies report that the antiproliferative effect of ENNs involves the inhibition of the cell cycle [8,48]. In HepG2 cells, ENN B1 (1.5 µM and 3 µM) increased the proportion of cells in the G_0_/G_1_ phase, leading to a decrease in proportion in the G_2_/M phase after 48 and 72 h of exposure [48]. Moreover, Prosperini et al. [51] showed that ENN B1 (ranging from 0.9 µM to 15 µM) arrested the cell cycle of CaCo-2 cells in the G_2_/M phase and the S phase after 24 h and 72 h of exposure, respectively. An alteration in the cell phases was also noted for an epithelial carcinoma-derived cell line (KB-3-1) treated with a mixture of ENNs (3% ENN A, 20% ENN A1, 19% ENN B, and 54% ENN B1), causing an increase in cells in the S phase after exposure to 2.5 μM and an increase in cells in the G_2_/M phase after exposure to 5 and 10 μM [48]. In addition to an impairment of the normal cell cycle, some studies suggest that ENN B1 could exert an embryotoxic effect [50,62]. Huang et al. [50], using mouse blastocysts, showed that exposure to ENN B1 (1–10 µM) led to negative effects on early-stage embryonic development and post-implantation development status through ROS-mediated apoptotic processes. Moreover, Wang et al. [47] reported that ENN B1 in concentrations ranging from 10 µM to 50 µM showed a negative effect on porcine embryo development by reducing cell division and blastocyst development rates. Indeed, exposure to ENN B1 led to alterations in the activity of DNA methyltransferases (*Dnmts*) and ten-eleven translocation (*Tet*) dioxygenases, which are essential for the normal expression of genes related to embryo development [47]. Specifically, ENN B1 led to the disruption of *Dnmt1*, *Dnmt3a*, *Tet1*, and *Tet3* transcription and an increase in the methylation level of centromeric satellite repeat (*CenRep*) and the pluripotent genes *Oct4*, *Nanog*, and *Sox2* [47].

### 4.5. Mitochondrial Membrane Permeabilization

Several studies have reported that the negative effects induced by ENN B1 on mitochondria are strongly connected to the ionophoric activity of this mycotoxin [8,21,36]. The possible mitochondrial toxicological activity was first investigated using intact mammalian cells (boar spermatozoa) by Hoornstra et al. [52], who found that exposure to 500 ng/mL (0.7 µM) of ENN B1 for 4 days blocked sperm motility by depolarizing the mitochondria and hyperpolarizing the plasma membrane of sperm cells. The effects of ENN B1 on mitochondrial function were confirmed using a mixture of ENNs (3% ENN A, 20% ENN A1, 19% ENN B, and 54% ENN B1), which induced a dose-dependent drop in the mitochondrial membrane potential (ΔΨm) in isolated rat liver mitochondria because of K^+^ influx into the mitochondrial matrix [36]. Moreover, the mixture of ENNs uncoupled the oxidative phosphorylation and suppressed the respiration rate, thus modifying the cellular homeostasis of rat liver mitochondria by causing damage to mitochondrial Ca^2+^ retention [36]. Lastly, its effects on mitochondria were reported using human Caco-2 cells where 24–74 h of exposure to ENN B1 (1.5–3 µM) induced a loss of mitochondrial membrane potential [51]. In contrast, no effects on mitochondrial membrane potential perturbation were recorded for HepG2 cells exposed to 1.5 µM and 3 µM of ENN B1 [46].

### 4.6. Genotoxicity

The genotoxicity of ENNs has been suggested by several authors [8,9,51]. A study by Prosperini et al., [51] highlighted the induction of DNA damage in Caco-2 cells exposed to ENN B1 concentrations ranging from 1.5 µM to 3 µM. Moreover, genotoxicity effects were evidenced with a comet test in HEK 293T cells after treatment with 25 µM of ENN B1 [54].

### 4.7. Estrogenic Activity

Recently, studies indicated that ENNs may act as endocrine disruptors in humans and wildlife [48,51,53]. However, to date, little is known regarding the estrogenic activity of ENN B1. A study performed by Park and Lee [53] using VM7Luc4E2 cells and following OECD Test Guideline (No.45)5 highlighted the capability of ENN B1 to act as an antagonist to the human estrogen receptor (ER) and androgen receptor (AR), with IC_50_ values of 6.76 × 10^−7^ M and 8.13 × 10^−7^ M, respectively. Additionally, it was shown that the mode of action (MoA) of the antagonistic ER/AR effects of ENN B1 was due to the inhibition of the dimerization of eRα/AR in cytosol [53].

### 4.8. In Vivo Toxicity

Currently, little information is available regarding the toxicological effects of ENN B1 in in vivo studies. Moreover, the few analyses available are connected to livestock [6,57,63]. Two pilot studies investigated the bioavailability of ENN B1 and showed it to be more prevalent in pigs (91% [62]) than in broiler chickens (5% [57]). Moreover, the EFSA Panel on Contaminants in the Food Chain (CONTAM [6]) identified no-observed-adverse-effect levels (NOAELs) for ENN B1 in broiler chickens (244 µg/kg bw/day) or laying hens (216 µg/kg bw/day). Callebaut et al. [63] showed the results of an 8 hr exposure to a mixture of ENNs, which demonstrated that ENN B1 did not alter the growth rate, feed uptake, and egg production of poultry. Additionally, Escriva et al. [64] reported that exposure to a mixture of ENNs (1.19, 2.16, 1.03, and 1.41 mg/kg body weight for ENN A, A1, B, and B1, respectively) did not induce observable adverse effects in Wistar rats after oral administration. In contrast, pregnant mice exposed to ENN B1 (5 mg/kg/d for 4 days) showed a decrease in the mRNA levels of innate immune-related genes as well as an increase in the ROS content and transcription levels of antioxidant enzymes [50]. Moreover, Kolf-Clauw et al. [59], using an ex vivo study, reported the intestinal toxicity of ENN B1. Specifically, concentrations ranging from 0.3 µM to 30 µM of ENN B1 led to a decrease in cell proliferation in pig tissue (jejunal explant) [59]. Lastly, an in vivo study by Huang and co-authors [50] confirmed embryonic cytotoxicity induced by ENN B1 for mice embryos with a 4-day intravenous injection of ENN B1 (1, 3, and 5 mg/kg body weight/d), leading to an increase in ROS levels and the apoptosis of the blastocyst-stage mouse embryos.

**Table 2:** ENN B1 toxicological effects.

Toxicological Effects	Models/Cells	Concentrations and Exposure Times	Effects	Ref.
	CaCo-2 cells	IC_50_ between 10.8 µM to 0.8 µM	Cytotoxic effects	[51]
	CaCo-2 cells	In total, 0.9 to 15.0 µM of binary, tertiary, and quaternary mixtures (ENN A, ENN A1, ENN B, ENN B1)	Cytotoxic effects	[45]
	HT-29 cells	IC_50_ between 16.6 µM and 3.7 µM	Cytotoxic effects	[28]
	IPEC-J2 cells	5 µM	Reduction in transepithelial electrical resistance	[55]
Cytotoxicity	HepG2 cells	IC_50_ between 24.3 µM and 8.5 µM	Cytotoxic effects	[56]
	MRC-5 cells	IC_50_ between 4.7 µM and 4.5 µM	Cytotoxic effects	[56]
	CHO-K1 cells	IC_50_ between 4.53 µM and 2.47 μM	Cytotoxic effects	[48]
	CHO-K1 cells	IC_50_ of 0.44 ± 0.15, ENN A1 + B mixture; IC_50_ of 0.97 ± 0.48, ENN A1 + B + B1 mixture.	Cytotoxic effects	[46]
	Insect SF-9 cells	IC_50_ of 6.6 µM	Cytotoxic effects	[34]
	PK-15 cells	IC_50_ of 41 µM	Cytotoxic effects	[35]
	CaCo-2 cells	1.5 and 3 µM	ROS overproduction Oxidative damage	[51]
Oxidative stress	Mouse blastocysts	1–10 µM	ROS overproduction	[60]
	Wistar rats	Mixture of ENNs (ENN A, ENN A1, ENN B and ENN B1)	ROS overproduction	[49]
Apoptosis	Pig embryos	10, 25, and 50 μM	Destruction of the anti-apoptosis signaling pathway	[47]
	SH-SY5Y cells	0.1 µM and 10 µM	Caspase-induced apoptotic cell death	[47]
	HepG2 cells	1.5 µM and 3 µM	Increase in the G0/G1 phase (48 h) and decrease in the G2/M phase (72 h)	[48]
Impairment of cell cycle	CaCo-2 cells	0.9 µM to 15 µM	Arrested the cell cycle at the G2/M phase (24 h) and S phase (72 h)	[51]
	KB-3-1 cells	mixture of ENNs (3% ENN A, 20% ENN A1, 19% ENN B, and 54% ENN B1)	Increase in cells in S phase and increase in cells in G2/M phase	[48]
	Mouse blastocysts	1–10 µM	Negative effects on early-stage embryonic development	[50]
	Porcine embryo	10 µM to 50 µM	Negative effects on early-stage development	[47]
	Boar spermatozoa	0.7 µM	Blocked sperm motility	[52]
Mitochondrial membrane permeabilization	Rat liver mitochondria	Mixture of ENNs (3% ENN A, 20% ENN A1, 19% ENN B, and 54% of ENN B1)	Drop in mitochondrial membrane potential (ΔΨm) Modification of the cellular homeostasis	[36]
	Caco-2 cells	1.5–3 µM	Loss of mitochondrial membrane potential	[51]
Genotoxicity	Caco-2 cells	1.5 µM to 3 µM	DNA damage	[51]
	HEK 293T cells	25 µM	DNA damage	[54]
Estrogenic activity	VM7Luc4E2 cells	IC_50_ = 6.76 × 10^−7^ M	Antagonist to the human estrogen receptor (ER)	[53]
	VM7Luc4E2 cells	IC_50_ = 8.13 × 10^−7^ M	Antagonist to the human androgen receptor (AR)	[53]

## 5. Conclusions and Future Challenges

Although specific data on emerging mycotoxins are limited, the distribution and occurrence of ENN B1 and ENNs in general, as already reported for other traditional fusariotoxins, are susceptible to climate change [12,65,66], and the need for a comprehensive overview of the contamination of non-regulated mycotoxins (for this reason, less investigated) is urgent. There is a scarcity of toxicokinetic and metabolism studies focused on ENN B1 [9,44,56,62,67,68] showing interesting species-specific differences in ENN B1 bioavailability [9,61], suggesting the need to collect more data from other species. Moreover, the few data on ENN B1′s carryover rate in animal products, even if generally low, showed the high presence of this mycotoxin in some tissues of turkeys, broilers [1,68,69], and farmed fish [70]. All these considerations suggest the need to expand kinetic and carryover studies to other species specifically related to ENN B1; furthermore, from a broader one-health point of view, considering that ENNB1 is an environmental toxin, other investigative approaches [48] should be applied. Moreover, little is still known regarding the possible estrogenic activity of ENN B1, and further studies should be a priority. The peculiarities of ENN B1 reported in this review suggest further specific studies on this emerging mycotoxin are needed, but even more importantly, this highlights the differences between various ENNs, an important aspect that must be considered for correct risk assessment and final regulations.
